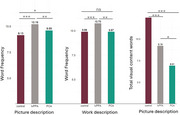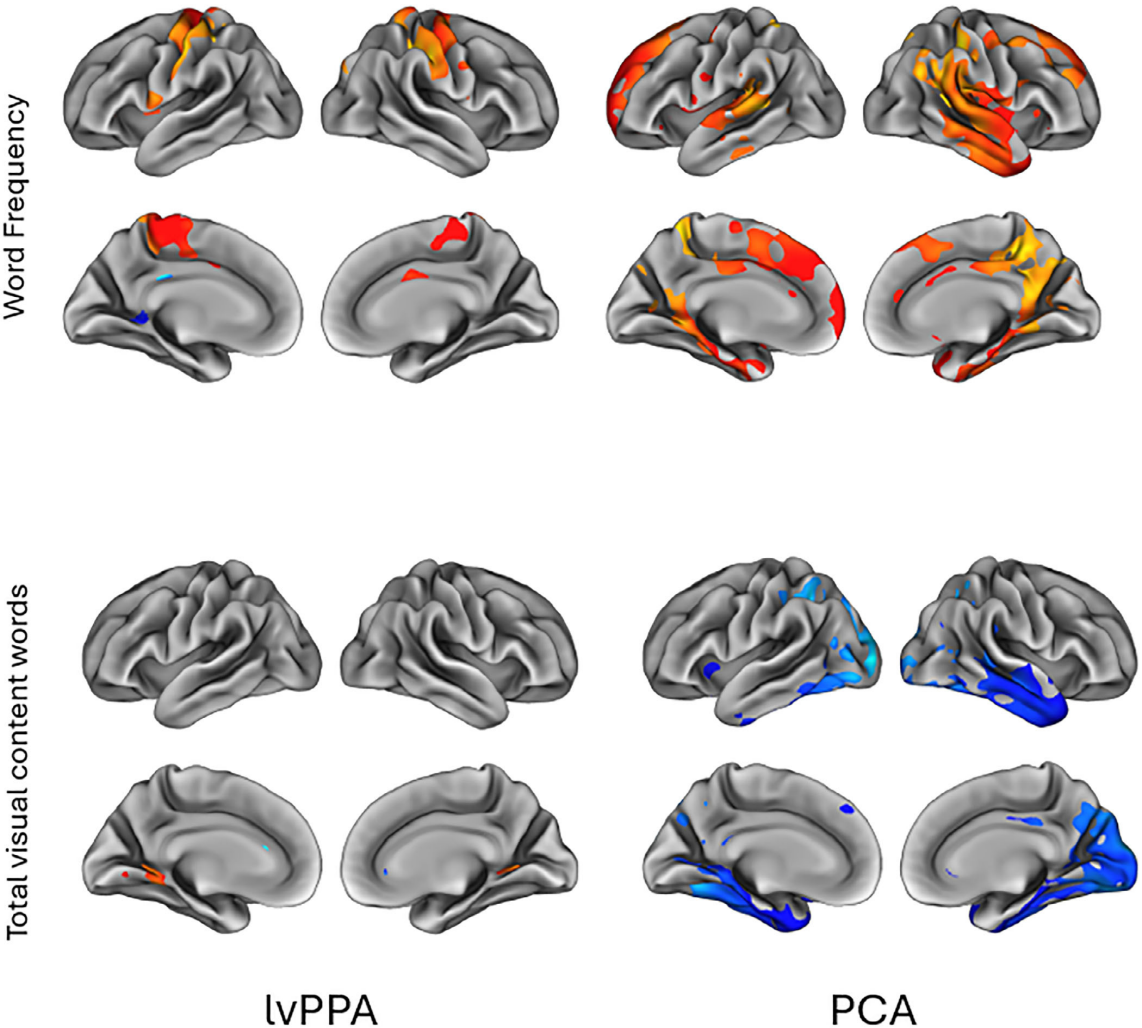# Digital Language Markers Differentiate Atypical Alzheimer's Disease Variants: Insights into Cognitive and Anatomical Mechanisms

**DOI:** 10.1002/alz70856_098249

**Published:** 2025-12-24

**Authors:** Neguine Rezaii, Yuta Katsumi, Daisy Hochberg, Nneka Watson, Megan Quimby, Brad C. Dickerson, Deepti Putcha

**Affiliations:** ^1^ Massachusetts General Hospital, Harvard Medical School, Boston, MA, USA; ^2^ Massachusetts General Hospital and Harvard Medical School, Boston, MA, USA; ^3^ Frontotemporal Disorders Unit and Massachusetts Alzheimer's Disease Research Center, Department of Neurology, Massachusetts General Hospital and Harvard Medical School, Boston, MA, USA

## Abstract

**Background:**

The computational analysis of language has demonstrated significant diagnostic value in typical older‐onset Alzheimer's disease (AD). Here, we investigate whether digital language markers can distinguish between variants of atypical AD, including logopenic variant Primary Progressive Aphasia (lvPPA) and Posterior Cortical Atrophy (PCA).

Both lvPPA and PCA patients exhibit deficits in spontaneous speech, such as difficulty accessing low‐frequency words. However, these deficits likely arise from distinct mechanisms: lvPPA patients have an intrinsic deficit in lexicosemantic retrieval, while deficits in PCA may be secondary to visual processing abnormalities. We hypothesize that distinct digital language markers can differentiate between these variants and provide insight into these cognitive mechanisms.

**Methods:**

We analyzed the spoken language of 29 healthy controls, 52 lvPPA participants, and 32 PCA participants during two tasks: 1) a picture description task requiring a high visual demand and 2) a job description task with minimal visual demand. Computational methods quantified word frequency and the total number of visual content words retrieved from the picture. Tau PET imaging was used to investigate the anatomical correlates of digital language markers in the picture description task.

**Results:**

Both lvPPA and PCA participants demonstrated difficulty accessing low‐frequency words during the picture description task. In the job description task, lvPPA participants continued to struggle to access low‐frequency words while PCA participants were comparable to healthy controls. Furthermore, although both AD variants retrieved fewer visual content words from the picture compared to healthy controls, PCA participants produced significantly fewer words, underscoring their challenges in processing visual information.

We found that word frequency positively correlated with tau deposition in distinct regions in lvPPA and PCA during the picture description task. Furthermore, the total number of visual content words was found to anti‐correlate with tau deposition in occipital visual processing areas in PCA but not in lvPPA.

**Conclusion:**

While both lvPPA and PCA patients struggle with low‐frequency word retrieval, this deficit in lvPPA stems from intrinsic lexicosemantic impairments, whereas in PCA, it is secondary to difficulties in visual processing. These results highlight the significant utility of digital language markers in differentiating between AD variants and understanding underlying language mechanisms.